# Cold vs. Room Temperature: A Comparative Analysis of Platelet Functionality in Cold Storage

**DOI:** 10.3390/biomedicines13020310

**Published:** 2025-01-27

**Authors:** Panagiotis V. Drossos, Sotirios P. Fortis, Alkmini T. Anastasiadi, Efthymia G. Pavlou, Andreas G. Tsantes, Gerasimos A. Spyratos, Effie G. Papageorgiou, Efrosyni G. Nomikou, Konstantinos E. Stamoulis, Georgios Dryllis, Vassilis L. Tzounakas, Marianna Politou, Serena Valsami, Anastasios G. Kriebardis

**Affiliations:** 1 Laboratory of Reliability and Quality Control in Laboratory Hematology (HemQcR), Department of Biomedical Sciences, School of Health & Caring Sciences, University of West Attica (UniWA), 12243 Egaleo, Greece; pdrossos@uniwa.gr (P.V.D.); sfortis@uniwa.gr (S.P.F.); pavlou@hippocratio.gr (E.G.P.); andreas.tsantes@yahoo.com (A.G.T.); makisspyratos@hotmail.com (G.A.S.); efipapag@uniwa.gr (E.G.P.); gdryllis@uniwa.gr (G.D.); 2 Department of Biochemistry, School of Medicine, University of Patras, 26504 Patras, Greece; aanastasiadi@upatras.gr (A.T.A.); vtzounakas@upatras.gr (V.L.T.); 3 Blood Bank and Hemophilia Unit, Hippokration Hospital, 11527 Athens, Greece; efrosyni.nomikou@gmail.com; 4 Laboratory of Haematology and Blood Bank Unit, Attikon University Hospital, School of Medicine, National and Kapodistrian University of Athens, 12462 Athens, Greece; 5 Hellenic National Blood Transfusion Centre, 13677 Acharnes, Greece; kostas.stamoulis@gmail.com; 6 Hematology Laboratory—Blood Bank, Aretaieion Hospital, National and Kapodistrian University of Athens, 11528 Athens, Greece; mariannapolitou@gmail.com

**Keywords:** cold-stored platelets, platelet functionality, ROTEM^®^ analysis, storage conditions

## Abstract

**Background:** The platelet functionality of cold-stored platelets remains a subject of debate. Our aim was to investigate the effect of temperature on the hemostatic properties of stored platelets. **Methods:** Ten split pooled platelets stored at cold and at room temperature were evaluated in vitro on storage days 1, 5, 10, and 15 for metabolic, physiological, and vesiculation parameters, as well as their hemostatic profile using rotational thromboelastometry (ROTEM^®^). **Results:** The integrity profile was better preserved in the cold-stored platelets, as lower lactate dehydrogenase levels were documented (e.g., day 10: 261 ± 46 vs. 572 ± 220 U/L, 4 vs. 22 °C, *p* = 0.004). A time-dependent decrease in hemostatic capacity was evident regardless of the temperature, but the cold-stored units were linked to shorter clot initiation times and increased elasticity, strength, and firmness parameters, especially during extended storage (e.g., maximum clot firmness, INTEM day 15: 81 ± 2 vs. 19 ± 4 mm, 4 vs. 22 °C, *p* = 0.0008). Additionally, the aggregation of cold-stored platelets was superior after the addition of any agonist tested. Regarding vesiculation parameters, the extracellular vesicles of the units at 4 °C were characterized by a larger size from day 10 onwards, when they also presented higher procoagulant activity (e.g., phospholipid-dependent clotting time of day 15: 21.4 ± 2.3 vs. 25.0 ± 3.0 s, 4 vs. 22 °C, *p* = 0.016). **Conclusion:** Our results indicate that cold-stored platelets perform better than those stored at room temperature, demonstrating superior clot formation and stability. This suggests that cold storage may more effectively preserve platelet function, potentially offering advantages for transfusion therapy and the extension of shelf-life. However, the clinical relevance of these findings requires further investigation.

## 1. Introduction

Platelet transfusion is a critical component of the treatment strategy for various bleeding disorders, including thrombocytopenia and trauma-induced hemorrhage. However, the effectiveness of these transfusions can be significantly affected by the storage conditions. Traditionally, platelets are stored at room temperature (RT), which is associated with a progressive decline in platelet function over time. This time-dependent loss of functionality can result in diminished hemostatic efficacy, posing challenges for the management of patients with severe bleeding [1].

Recent research has proposed cold storage (CS), typically at 4 °C, as a potential alternative to RT storage. CS has been shown to better preserve platelet function and extend their shelf life [2,3,4]. The benefits of CS are attributed to its ability to reduce platelet activation and apoptosis, which are common issues in RT-stored platelets. By maintaining platelet stability and minimizing the degradation of crucial platelet components, CS can enhance their hemostatic properties and potentially improve transfusion outcomes [3,5,6].

Moreover, the CS of platelets offers several important benefits, particularly in reducing the risk of bacterial contamination [7]. Since platelets are typically stored at RT, they are more susceptible to bacterial growth [8], which can lead to transfusion-related infections [9]. CS inhibits bacterial proliferation, thereby enhancing the safety of transfusions [10]. Additionally, CS has been shown to better preserve platelet function [2], making them more effective in various clinical settings. These advantages indicate CS as a promising method for improving the efficacy of platelet transfusions [11].

Despite these potential benefits, there remains a need for a thorough evaluation of CS’s impact on platelet functionality, especially after long storage. The current research suggests that CS for a long period (10 and 15 days) may preserve platelet functionality more effectively than RT storage. This study aims to systematically compare CS-platelets with RT-platelets across various parameters, including clot formation, platelet stability, and overall hemostatic efficacy. By conducting this comparative analysis, we seek to provide valuable insights into the advantages and limitations of CS, potentially guiding future improvements in platelet transfusion practices and enhancing patient outcomes.

## 2. Materials and Methods

### 2.1. Pooled Platelet Production

A total of 450 ± 50 mL of whole blood was collected in Reveos bags from volunteers meeting the European blood donation criteria according to the Hellenic National Blood Transfusion Centre. The blood bags were kept overnight at 22 ± 2 °C after blood collection (Day 0). Whole blood-derived platelet-rich plasma was prepared using soft-spin centrifugation in an automated blood processing system, yielding 57 mL autologous plasma. Each pooled platelet unit (volume 285 mL) consisted of five platelet-rich plasma units. After pooling, the platelets were leukoreduced using the pooling set kit (Terumo, Tokyo, Japan). The five pooled and filtered platelet-rich plasmas contained sufficient plasma for storage and did not require the addition of a platelet medium. Initially, ten pooled platelet units (Day 1) were produced (Blood group ABO, O *n* = 3, B *n* = 2, AB *n* = 2, A *n* = 3). Afterward, the units were split into two sub-units: half were agitated at 4 °C in a cold room (CS-platelets) and half at 22 °C (RT-platelets). On storage days 1 (baseline assessment), 5, 10, and 15, platelet samples were collected aseptically, and analysis was completed immediately. All the experiments were performed in the total number of samples (*n* = 10 per group) at all time points.

### 2.2. Culture of Platelets

Small volumes of platelets were cultured aerobically and anaerobically as described by the FDA [12]. For sampling on days 5 and 15 of storage, 0.1 mL aliquots were inoculated onto blood agar plates. These plates were then incubated in a 5% CO_2_ atmosphere for up to 48 h. Additionally, anaerobic cultures were performed by incubating a second set of plates in an anaerobic environment. No microbial growth was observed in any of the platelet cultures.

### 2.3. Biochemical and Hematology Analysis

Biochemical analyses of the supernatant plasma were performed using an Architect Clinical Chemistry Analyzer (Abbott Laboratories, Abbott Park, IL, USA). General blood tests were performed using a Siemens Advia 2120i Hematology Analyzer (Basel, Switzerland).

### 2.4. Measurement of Intracellular Reactive Oxygen Species Levels in Platelets

The intracellular levels of ROS (iROS) were detected with the membrane-permeable fluorescent probe CMH_2_DCFDA (Invitrogen, Molecular Probes, Eugene, OR, USA). Stored platelets were obtained (200× *g* for 20 min), diluted in HEPES Tyrode’s buffer containing 0.1% bovine serum albumin (BSA), and incubated with 10 μM CMH_2_DCFDA for 30 min at 37 °C in the dark. The mean fluorescence index was determined by flow cytometry in a FACSCanto II Cytometer (BD Biosciences, San Jose, CA, USA). Data analysis was performed using the BD FACSDiva™ Software (Version 6.0) [13].

### 2.5. Plasma Phospholipid Procoagulant Activity Assay

PPL activity was assessed using the STA^®^–Procoag-PPL kit (STAGO, Paris, France) using the STAR MAX^3^ STAGO analyzer according to the manufacturer’s protocol. The time to clot formation or other relevant coagulation endpoints was recorded in seconds. Short coagulation time indicates high PPL activity [14].

### 2.6. P-Selectin Expression and Phospatidylserine Exposure

Briefly, the cold and room temperature platelets diluted in Annexin V-binding buffer (Hepes/NaOH 0.01 M, NaCl 140 mM, CaCl_2_ 2.5 mM) were incubated 15 min in room temperature with allophycocyanin (APC)-conjugated anti-CD62P and PE-Annexin V along with the platelet gating marker PE Cy5-CD41a (BD Biosciences, San Jose, CA, USA). Reactions were stopped with Annexin V-binding buffer and measurements were performed by flow cytometry [13,15].

### 2.7. Nanoparticle Tracking Analysis

A nanoparticle tracking analysis (NTA) was conducted using the NanoSight NS300 instrument (Malvern Instruments Ltd., Malvern, UK). For analysis, the platelet-poor plasma (PPP) was diluted in particle-free PBS (0.02 μm filtered, Cytiva Whatman, Maidstone, UK). Autofocus was adjusted to ensure clarity by avoiding indistinct particles. Five 30 s videos were recorded per measurement under the following conditions: cell temperature at 25 °C and syringe speed at 100 μL/s. The videos were analyzed using the NanoSight NTA 3.4 build 3.4.4 software in script control mode, with a total of 1500 frames per sample. Each sample was measured five times, and the size distribution data were averaged [16].

### 2.8. Rotational Thromboelastometry

The viscoelastic properties of the platelet concentrates were evaluated using an ROTEM analyzer (Tem Innovations GmbH, Munich, Germany). The cold and room temperature-stored platelets were tested according to the manufacturer’s instructions for whole blood. The ROTEM^®^ analysis included the EXTEM, INTEM, and FIBTEM assays. For the EXTEM assay, clot formation was induced by activating the extrinsic coagulation pathway by adding tissue thromboplastin, while for the INTEM assay, the intrinsic pathway was activated by ellagic acid. The FIBTEM assay, a modified EXTEM, includes the addition of cytochalasin D, which blocks platelets’ activation, shape change, and the expression/activation of glycoprotein IIb/IIIa (fibrinogen) receptors. We measured the FIBTEM to calculate the platelet-specific ROTEM^®^ (PLTEM) by subtracting the FIBTEM from the EXTEM.

### 2.9. Platelet Aggregation

The platelet count of the cold and RT samples was adjusted between 200 × 10^9^/L and 300 × 10^9^/L with the PPP. The pooled platelet concentrates were centrifuged (2000× *g*/15 min) to obtain the PPP [17]. The PPP was used to set a 100% upper limit and pooled stored platelets to set a 0% baseline before the addition of the agonist. A panel of 5 different agonists was used as follows: collagen (5 μg/mL), ADP (5 μΜ), arachidonic acid (1.5 U/mL), epinephrine (15 μΜ), and Restocetine (0.5 and 1.2 μg/mL) according to the manufacturer’s instructions (Helena, BioSciences Europe, Gateshead, UK). Aggregation was performed using an AggRAM aggregation remote analyzer module.

### 2.10. Statistical Analysis

Data are expressed as mean ± standard deviation (SD). All the parameters were tested for normal distribution profile using detrended Q-Q plots and Shapiro–Wilk test, as well as the presence of outliers. Between-group differences were evaluated by repeated measures ANOVA with Bonferroni-like adjustments for multiple comparisons after checking for the appropriate assumptions. Statistical significance was accepted at a *p* < 0.05. In the case of extreme outliers, the aberrant value was excluded and the test was performed again. If the outcome of the analysis remained the same, the outlier was included in the final dataset. Statistical analysis was carried out with the IBM SPSS Software (version 27.0 for Windows IBM Corp., Armonk, NY, USA; administrated by UniWA—University of Patras).

## 3. Results

### 3.1. Platelet Physiology–Biochemistry

**Figure 1:**
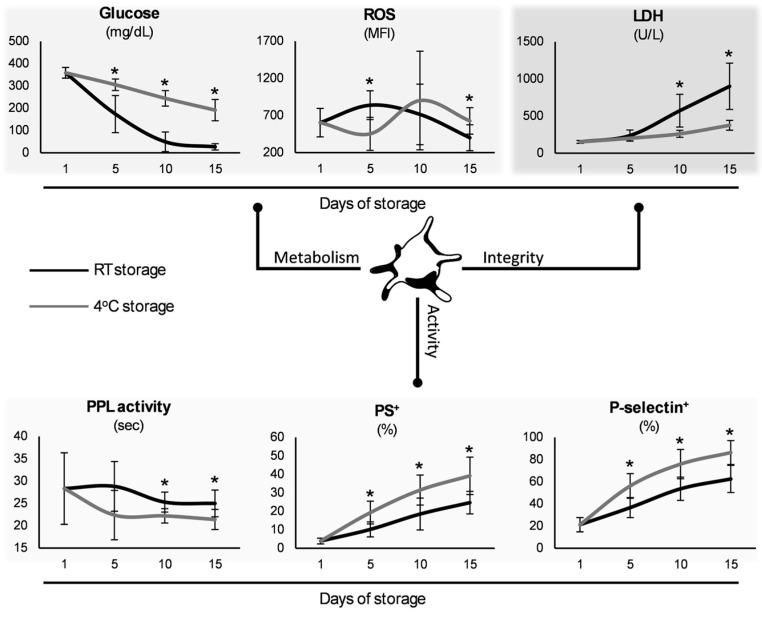
Metabolic, integrity, and activity parameters of cold-stored versus conventionally stored platelets (*n* = 10 per group). ROS: reactive oxygen species; LDH: lactate dehydrogenase; RT: room temperature; PPL: procoagulant phospholipid; PS: phosphatidylserine. (*) *p* < 0.05.

In both the CS-platelets and RT-platelets, glucose consumption significantly increased over time (*p* < 0.05), with the CS-platelets showing lower rates at all the time points (Figure 1). ROS accumulation was lower in the CS-platelets at day 5 in comparison to the RT-platelets, but the opposite motif emerged at the last time point. While in both groups the concentrations of lactate dehydrogenase (LDH) elevated over time, the CS-platelets presented significantly lower values when stored for 10 or more days. Since the RT-platelets’ late time points have limited clinical relevance, a comparison was also made between days 10 and 15 of cold storage and day 5 in RT. Notably, the LDH levels of the CS-platelets exceeded those of the 5-day-stored RT-platelets only on day 15 (*p* = 0.0007). It should also be noted that while no difference was observed between the groups in PLT count, the time-course drop in the levels was evident earlier on in the cold group (e.g., 2263 ± 210 vs. 2394 ± 201 × 10^3^/μL, day 10 CS-platelets vs. baseline, *p* = 0.0022), while it only exceeded the threshold for statistical significance in the final time point of their counterparts (2244 ± 268 vs. 2394 ± 201 × 10^3^/μL, day 15 RT-platelets vs. baseline, *p* = 0.048). Regarding surface markers, phosphatidylserine (PS) exposure increased almost linearly during storage and was significantly higher in the CS-platelets versus the RT-platelets throughout the storage period (Figure 1). A similar finding emerged regarding the expression of P-selectin. Since activated platelets can produce microvesicles enriched in phospholipids, the phospholipid-dependent procoagulant activity was assessed. The clotting time was shortened throughout storage in hypothermic conditions, with the significance exceeding the threshold of multiple comparisons in the last two time points (Figure 1). On the other hand, the presence of thrombin–antithrombin complex, a useful indicator of thrombin levels, was similar between the two groups (e.g., day 5: 6.84 ± 3.59 vs. 6.58 ± 1.94 mg/L, CS-platelets vs. RT-platelets, *p* = 0.737). No microbial growth was observed in any of the platelet cultures.

### 3.2. Vesiculation

**Figure 2:**
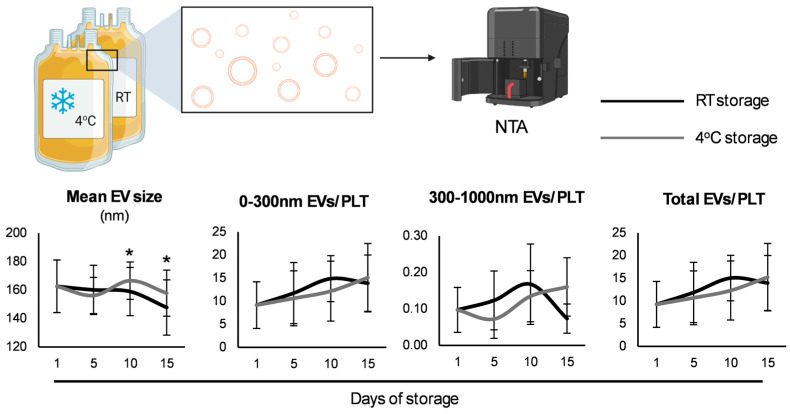
Vesiculation parameters of cold-stored versus conventionally stored platelets (*n* = 10 per group) using nanoparticle tracking analysis (NTA). RT: room temperature; EV: extracellular vesicles; PLT: platelet. (*) *p* < 0.05.

It is well known that during storage, the alterations that occur to the platelet membrane result in the production of extracellular vesicles (EVs) [18]. Interestingly, no difference was observed between the two storage strategies regarding vesiculation rates; yet, the mean size of EVs was higher in the case of the CS-platelets beyond the 5 days of the RT-platelets (Figure 2).

### 3.3. Hemostatic Parameters

**Figure 3:**
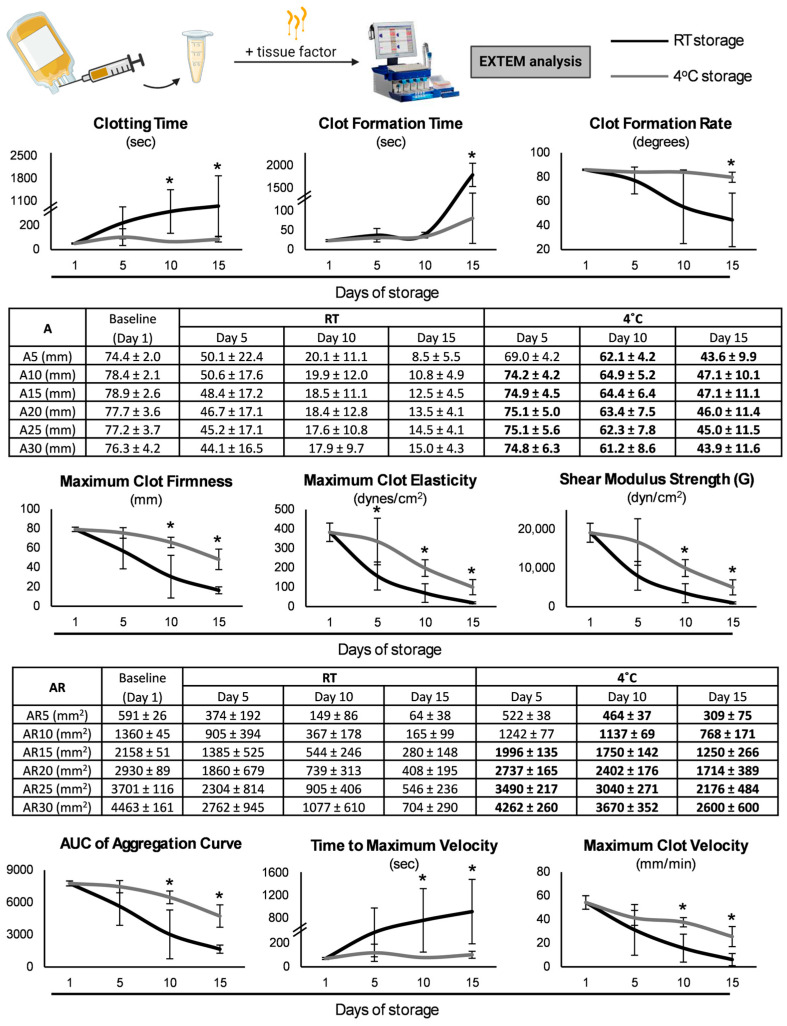
Assessment of the viscoelastic properties of the cold-stored versus conventionally stored platelets (*n* = 10 per group) after the addition of tissue factor (EXTEM assay). RT: room temperature; Ax: amplitude x minutes after clotting time (CT); ARx: area under the curve (AUC) from CT to x minutes. (*), numbers in bold: *p* < 0.05.

**Figure 4:**
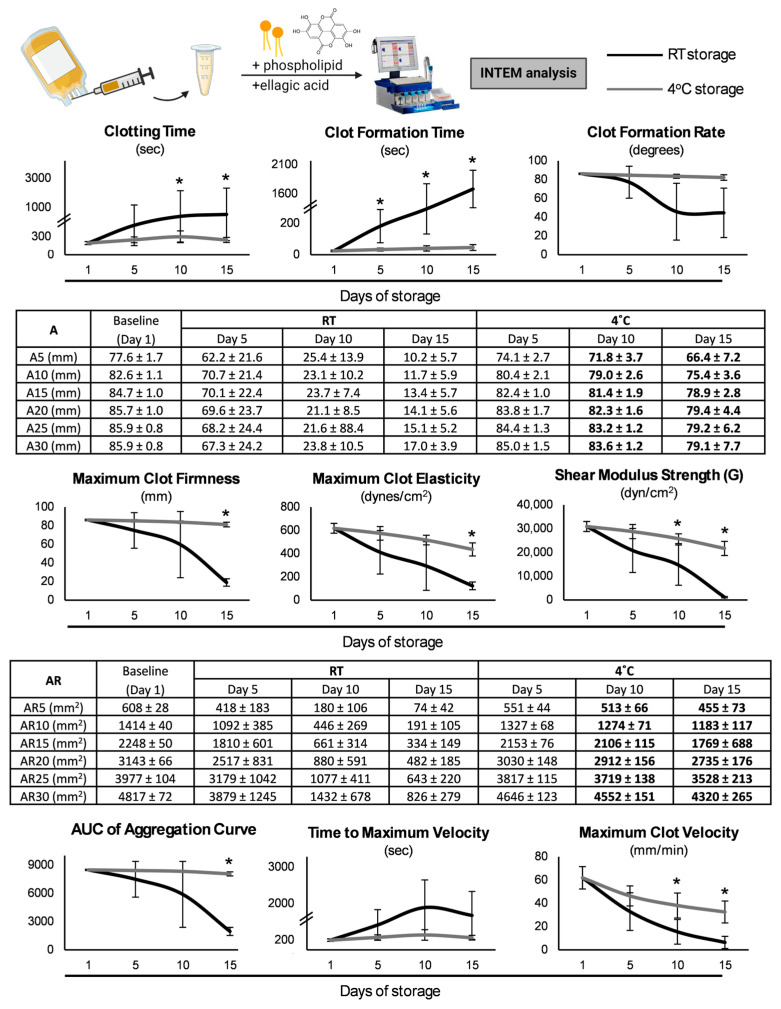
Assessment of the viscoelastic properties of the cold-stored versus conventionally stored platelets (*n* = 10 per group) after coagulation activation via the contact phase (INTEM assay). RT: room temperature; Ax: amplitude x minutes after clotting time (CT); ARx: area under the curve (AUC) from CT to x minutes. (*), numbers in bold: *p* < 0.05.

Upon the induction of the coagulation cascade either via the extrinsic (Figure 3) or the intrinsic (Figure 4) pathway, extensive differences emerged between the two storage strategies. Regarding the initiation of coagulation, clotting time maintained low levels at 4 °C, a finding highlighted by the lower levels observed on day 15 even when compared to the RT-platelets of day 5 (e.g., INTEM: 236 ± 40 vs. 470 ± 323 s, day 15 of CS vs. day 5 of RT-stored, *p* = 0.047). Additionally, the clot formation time was minor in hypothermic conditions either at the end (EXTEM) or throughout (INTEM) the storage period. The results of the kinetics of clot formation were also interesting, with the CS-platelets showing increased amplitude values from 5 to 30 min after clot initiation either for all the time points (EXTEM assay) or from day 10 onwards (INTEM assay). This superiority was also preserved until reaching maximum clot firmness (MCF), where the values were greater in the case of the CS-platelets for days 10 and 15 after tissue factor addition, and for day 15 after the initiation of the intrinsic pathway. Similarly, the two values that better represent clot strength, namely maximum clot elasticity (MCE) and shear modulus strength (G), were found elevated either throughout storage (EXTEM) or mainly at the end of it (INTEM) in the same group. Notably, especially regarding INTEM, on day 15, the CS-platelets exhibited values similar to the ones of their counterparts on day 5 (e.g., MCE: 436 ± 56 vs. 411 ± 187 dynes/cm^2^, day 15 of CS vs. day 5 of RT-stored, *p* = 0.710). Regarding velocity, maximum velocity was higher in the two last time points in the CS-platelets upon coagulation initiation either intrinsically or extrinsically, while a shorter time (or a statistical trend) was needed to reach this value in comparison to the RT-platelets. Area under the curve (AUC) values from 5 to 30 min after clot initiation were also significantly higher throughout storage (EXTEM) or beyond the fifth day (INTEM) in the CS-platelets (Figure 3 and Figure 4).

When the platelet-specific ROTEM^®^ (PLTEM) was calculated by extracting FIBTEM from EXTEM, significant results emerged regarding the contribution of platelets. For instance, the increased platelet contribution in the strength of the clot in CS became evident given the values of MCE throughout its duration, already starting from day 5 (295 ± 111 vs. 125 ± 73 dynes/cm^2^, CS vs. RT-stored, *p* = 0.034). The same pattern, with greater values in the group of hypothermic storage, was observed regarding the other firmness-related parameters of PLTEM, such as G and MCF (e.g., G of day 5: 14,684 ± 5543 vs. 6347 ± 3749 dynes/cm^2^, CS vs. RT-stored, *p* = 0.036). It should be noted that the latter parameters presented similar levels to day 5 RT storage even when reaching day 15 in the cold group (e.g., MCF: 23.67 ± 7.10 vs. 32.51 ± 13.91 mm, day 15 of CS vs. day 5 of RT-storage, *p* = 0.23).

### 3.4. Aggregation Profile

**Figure 5:**
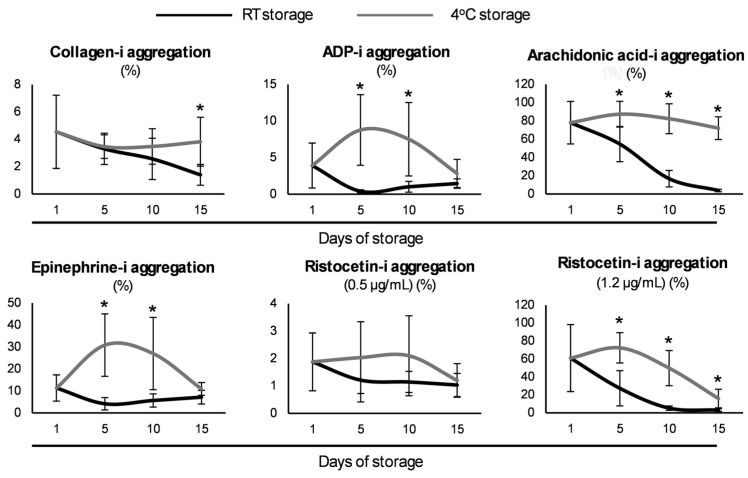
Aggregation of the cold-stored versus conventionally stored platelets (*n* = 10 per group) after induction by different agonists. The results on x-aris are in maximum percentage (max %) aggregation; RT: room temperature; ADP: adenosine diphosphate. (*) *p* < 0.05.

To evaluate the aggregation properties of the stored platelets, samples from the two storage strategies were exposed to several agonists to examine their response. When collagen was added, while the RT-platelets exhibited a time-dependent decrease in terms of aggregation, their CS counterparts presented more stable levels, something that emerged as statistically significant on day 15 (Figure 5). On the other hand, upon exposure to ADP or epinephrine, aggregation was greater in the same units from day 5 but reached control levels at the end of the storage period. Finally, universal differences emerged when in the presence of arachidonic acid or ristocetin (at high concentrations), again with the cold platelets being more responsive to the aggregation signal (Figure 5). When compared to the fifth day of the RT-stored platelets, the aggregation levels upon exposure to ADP, arachidonic acid, and epinephrine were higher even on days 10 (all three) and 15 (apart from epinephrine) in the cold group (e.g., arachidonic acid-i aggregation: 82 ± 6 vs. 55 ± 29%, day 10 in the cold vs. day 5 in RT, *p* = 0.013).

## 4. Discussion

The impact of CS on platelets was assessed over various time points to understand how prolonged refrigeration affects their characteristics and functionality. Our results indicate that CS-platelets demonstrate enhanced clotting capabilities, a finding that is in line with studies that focus on trauma and surgical patients [19,20].

### 4.1. Metabolism–Integrity–Activity

The lesser reduction in glucose levels observed in the CS-platelets suggests that while glucose metabolism is affected in both cases, CS has a greater impact on metabolic enzyme functionality [2,21,22]. At the same time, the fact that after the fifth day of storage, the CS-platelets exhibited lower levels of LDH indicates that CS might better preserve platelet integrity and reduce cellular damage over time, experiencing less cellular stress and degradation [22]. The CS-platelets exhibited lower ROS accumulation compared to the RT-platelets in early storage, contradicting the findings of Bynum et al. [23], who reported consistently lower mitochondrial ROS levels. Activated platelets produce ROS, inducing mitochondrial dysfunctions [24]. The observed reduction in ROS levels is noteworthy as elevated ROS can contribute to oxidative stress and platelet dysfunction [25]. After long storage, in agreement with Hegde et al. [26], the ROS generation was increased, a feature that may result in cytoskeletal and signaling impairment [27]. These findings suggest that the CS of platelets for up to 5 days may offer a beneficial alternative to conventional storage methods, particularly in scenarios where oxidative stress is a concern [26].

The increased presence of PS in the CS-platelets can be attributed to several biochemical and biophysical changes affecting platelet membranes. Cooling induces alterations in membrane fluidity, leading to a redistribution of membrane phospholipids [2], and promoting the exposure of PS on the platelet surface [28]. The higher prevalence of PS on the CS-platelets may impact their function and role in the coagulation process. On the other hand, P-selectin, a glycoprotein found in the alpha granules of platelets, is translocated to the surface in response to activation stimuli and plays a crucial role in platelet adhesion and interaction with other cells [22]. CS can induce platelet activation through various mechanisms, including alterations in membrane fluidity and the induction of cellular stress [29]. The increased surface expression of P-selectin in the CS-platelets is indicative of their activated state and can impact their function in thrombus formation and clotting processes.

### 4.2. Platelet Microvesiculation

Platelet-derived EVs are believed to mediate several hemostatic events along with platelets, and their concentration is reported to increase after CS [30], a finding that did not arise in the present study. After 10 days of storage, the CS-platelets exhibited larger EVs compared to those stored at RT, as previously described [31]. This finding suggests that CS conditions significantly impact the size of the EVs released by platelets. Larger EVs in the CS-platelets may express distinct markers, potentially altering downstream effects upon transfusion, including possible inflammatory responses. This has been suggested in older CS-platelets due to the release of biological response modifiers such as CD40L [32].

The observation that the CS-units exhibit shorter coagulation times (PPL assay) along with increased levels of PS and P-selectin highlights complex alterations in platelet functionality due to storage conditions, which could have clinical implications for situations requiring transfusion with highly procoagulant platelets. The increased PPL activity, along with the rise in PS, suggests a positive effect on the functional capacity of platelets and their EVs to contribute to coagulation [33]. Increased PPL activity and platelet activation could theoretically improve clot formation; however, if platelets are compromised by storage conditions, their contribution to effective hemostasis might be diminished [30,34].

### 4.3. Hemostatic Potential

We assessed the hemostatic potential of platelets using the ROTEM^®^ analysis, a method that provides an overall assessment of the coagulation mechanism, from clot formation and stabilization to clot retraction and breakdown [35]. The results of the INTEM and EXTEM assays highlight the superior hemostatic performance of the CS-platelets compared to the RT-platelets, particularly in the later stages of storage. Interestingly, although storage at RT has a negative time-dependent impact on the hemostatic capacity [35], the coagulation potential of the CS-platelets was not affected by the storage period, since these platelets maintained adequate hemostatic capacity even at the end of storage, hinting at the potential of an extended shelf-life for up to 15 days.

More specifically, the significant reduction in both clotting time and clot formation time in the CS-platelets suggests a more efficient and expedited initiation and development of clotting [36]. Moreover, the increased MCF and MCE indicate an enhanced clot strength and resistance to mechanical stress [37]. Overall, CS resulted in stronger clots that are more resistant to fibrinolysis, which could be attributed to increased thrombin formation because of cold temperature, and to the so-called cold-induced activation of platelets via cross-linking glycoprotein Ib or increased interaction with protein complexes [37]. In the same context, scanning electron microscopy analyses have shown that CS results in denser clots with thinner fibers and more crosslinks, a structure that could also provide higher stability [6]. Our findings regarding the faster formation of more robust clots in CS are in line with the results of Reddoch et al., who evaluated the impact of CS on the functional status of apheresis platelets using thromboelastography (TEG) [36]. The authors of this study found that clot strength, as reflected by the maximum amplitude, was better preserved at 4 °C, when clot formation was also faster, as shown by the reaction time. These TEG parameters are similar to MCF and clotting time in the ROTEM^®^ analysis, respectively, which were, likewise, affected by CS in our study. The increased shear modulus strength and higher AUC of the aggregation curve that was seen in the INTEM assay for the CS-platelets further support improved platelet functionality and aggregation capacity [38].

Since the superior hemostatic profile of the CS-platelets remained even upon the inhibition of platelets, coagulation factors may play a central role in this property by being better preserved in the cold. Nonetheless, the improved preservation of platelets is highlighted upon the calculation of PLTEM, suggesting that both cells and factors benefit from hypothermic temperatures. To support this, the RT-platelets showed a notable decline in aggregation capacity compared to the CS-platelets, a finding consistent with previous research indicating that RT leads to a progressive loss of platelet function due to increased activation and apoptosis [39]. Conversely, the CS-platelets exhibited enhanced aggregation responses, supporting the notion that CS better preserves platelet function and stability [19,40,41]. However, there are controversial studies that show a decrease in the survival of CS-platelets in comparison to RT-platelets [28,42]. When exposed to different activators, CS-platelets consistently demonstrated superior aggregation [2]. Specifically, collagen, ADP, and epinephrine elicited a more robust aggregation in CS-platelets compared to RT-platelets [43]. Collagen and ADP are known to activate platelets through distinct pathways: collagen triggers platelet activation via integrin receptors, with this activation causing the release of secondary agonists such as thromboxane A2 and the secretion of ADP from platelet-dense granules. A similar study showed that CS preserves these activation pathways more effectively, as an enhancement of induced platelet aggregation up to 30–40% was detectable, pointing to the onset of promoted reactivity within 1 h of storage at cold [44]. Epinephrine, which promotes platelet activation through adrenergic receptors, had a similar effect on CS-platelets. However, another study shows that the major disadvantage of CS is the clustering of GPIb on the platelet surface resulting in accelerated clearance from the circulation after re-transfusion by hepatic macrophages [45]. Arachidonic acid, which stimulates platelet activation via the cyclooxygenase pathway, and ristocetin, which activates platelets through the von Willebrand factor, also resulted in enhanced aggregation in CS-platelets. This suggests that CS minimizes the degradation of the key platelet components involved in these activation pathways, leading to low-level GPIIb-IIIa activation and resulting in aggregate formation over time. This chain reaction improves the metabolic and functional response of apheresis platelets in plasma while being stored in an additive solution at 4 °C [46]. Overall, the superior aggregation of CS-platelets across various activators supports the potential benefits of adopting CS protocols over traditional RT storage [47].

The higher coagulation potential that was observed across the ROTEM^®^ and aggregation assays for the CS-platelets suggests that CS significantly boosts platelet performance, offering critical advantages in clinical settings where rapid and robust hemostatic responses are essential. In trauma patients, for example, in whom post-transfusion platelet recovery is less of a concern, CS-platelets are a viable option to restore hemostasis [48].

### 4.4. Limitations

Despite presenting promising findings, this study has several limitations. Firstly, the analysis was conducted in a limited number of concentrates, thus underestimating the extensive variability seen in clinical practice. The study also focused primarily on in vitro assays, and while these provide valuable insights, they may not entirely reflect the complex interactions of platelets in a clinical setting.

## 5. Conclusions

This study demonstrates that CS-platelets exhibit superior functionality compared to room temperature-stored platelets, including enhanced clotting ability, better preservation of metabolic integrity, and reduced oxidative stress over time. The CS-platelets showed improved hemostatic potential, with faster clot formation, stronger clot resistance, and better preservation of platelet aggregation capacity even after extended storage. These findings suggest that CS-platelets may offer significant clinical advantages in transfusion therapy, particularly in trauma and surgical patients who require rapid hemostatic responses. The novelty of this study lies in its demonstration that CS-platelets can maintain optimal functionality for up to 15 days, providing a promising alternative to conventional platelet storage methods and potentially extending their shelf life without compromising efficacy. These results could inform the development of new platelet storage protocols that enhance both platelet performance and transfusion outcomes.

CS may more effectively preserve platelet function, potentially offering advantages for transfusion therapy and the extension of shelf-life. Future research should address these limitations by including a larger sample size and incorporating clinical outcome measures to validate the in vitro findings.

## Data Availability

The data presented in this study are available upon reasonable request.
